# Phosphorylation of S11 in PHR1 negatively controls its transcriptional activity

**DOI:** 10.1111/ppl.13831

**Published:** 2022-12-23

**Authors:** Ricardo Trejo‐Fregoso, Iván Rodríguez, Alejandra Ávila, Javier Andrés Juárez‐Díaz, Rogelio Rodríguez‐Sotres, Eleazar Martínez‐Barajas, Patricia Coello

**Affiliations:** ^1^ Departamento de Bioquímica Facultad de Química, UNAM. Cd. Mx Mexico City Mexico; ^2^ Departamento de Biología Comparada Facultad de Ciencias, UNAM. Cd. Mx Mexico City Mexico

## Abstract

Plant responses to phosphate starvation (−Pi) are very well characterized at the biochemical and molecular levels. The expression of thousands of genes is modified under this stress condition, depending on the action of Phosphate starvation response 1 (PHR1). Existing data indicate that neither the *PHR1* transcript nor the quantity or localization of its protein increase during nutrient stress, raising the question of how its activity is regulated. Here, we present data showing that SnRK1 kinase is able to phosphorylate some phosphate starvation response proteins (PSRs), including PHR1. Based on a model of the three‐dimensional structure of the catalytic subunit SnRK1α1, docking simulations predicted the binding modes of peptides from PHT1;8, PHO1 and PHR1 with SnRK1. PHR1 recombinant protein interacted in vitro with the catalytic subunits SnRK1α1 and SnRK1α2. A BiFC assay corroborated the in vivo interaction between PHR1 and SnRK1α1 in the cytoplasm and nucleus. Analysis of phosphorylated residues suggested the presence of one phosphorylated site containing the SnRK1 motif at S11, and mutation in this residue disrupted the incorporation of ^32^P, suggesting that it is a major phosphorylation site. Electrophoretic mobility shift assay results indicated that the binding of PHR1 to P1BS motifs was not influenced by phosphorylation. Importantly, transient expression assays in Arabidopsis protoplasts showed a decrease in PHR1 activity in contrast with the S11A mutant, suggesting a role for Ser11 as a negative regulatory phosphorylation site. Taken together, these findings suggest that phosphorylation of PHR1 at Ser11 is a mechanism to control the PHR1‐mediated adaptive response to −Pi.

## 1 INTRODUCTION

Phosphate starvation (−Pi) is a stress condition that occurs when sufficient Pi cannot be acquired from the soil. Since Pi is required for the synthesis of cellular molecules, in particular ATP, Pi deficiency leads to low‐energy conditions and metabolic impairment, activating different signaling pathways (Chiou & Lin, 2011). Several types of adaptive responses have been documented to promote Pi acquisition, including changes in root architecture, increased expression of high‐affinity Pi transporters to improve Pi uptake, enhanced Pi recycling from intra‐ and extracellular pools and metabolic adaptations to overcome the need for P‐containing intermediates in metabolic pathways (Bechtaoui et al., 2021). All of these responses are part of local and systemic signaling (Ham et al., 2017).

During Pi starvation responses, the expression of a large number of genes is altered (Bustos et al., 2010) under the control of key transcription factors (TFs) (Jain et al., 2012). Phosphate response 1 (PHR1) is a MYB domain family of TFs that are able to recognize P1BS elements (5'‐GNATATNC‐3′) in gene promoters (Sega & Pacak, 2019). In addition, a small subfamily of MYB domain TFs, highly related to PHR1, have been identified. The members of this MYB subfamily in Arabidopsis include PHR1 and four PHR 1‐like 1 TFs (PHL1, PHL2, PHL3, and PHL4). Even though PHR1 is the major TF modulating the transcriptional response to Pi deficiency, other members of the subfamily may play partially redundant roles, particularly PHL1 and PHL2 (Barragán‐Rosillo et al., 2021). The activity of PHR1 under −Pi conditions does not depend on transcript or protein accumulation (Puga et al., 2014). Early events in Pi starvation signaling allowed the identification of related proteins in yeast containing an SPX domain. The SPX domain was named after the suppressor of yeast *gpa1* (Syg1), the yeast cyclin‐dependent kinase inhibitor (Pho81) and the human xenotropic and polytropic retrovirus receptor 1 (XPR1) and comprises a group of amino acids with conserved lysine residues. The Arabidopsis genome contains 20 genes encoding proteins with SPX domain, 4 of which form a unique subfamily named *AtSPX1‐AtSPX4*. Analysis of the expression of *SPX1‐SPX4* in *phr1* mutants suggests that AtSPX1 and AtSPX2 are upstream regulators of PHR1 during Pi signaling (Duan et al., 2008). SPX1 interacts in vitro and in vivo with PHR1, and according to current evidence, the interaction of PHR1 with SPX domain proteins might regulate its translocation to the nucleus and prevent its direct binding to the promoters of target genes (Jung et al., 2018; Puga et al., 2014). Interestingly, the formation of this complex is stabilized by inositol‐pyrophosphates (PPi‐InsPs), and mutant plants unable to biosynthesize InsP8 exhibit constitutive activation of phosphate starvation‐induced genes (*PSI*; Dong et al., 2019). Therefore, even though PHR1 cannot be directly modulated by Pi, its activity can be fine‐tuned by the formation of an SPX‐InsP complex in a Pi availability‐dependent manner. PHR1 is also modulated by posttranslational modifications (PTMs), which might alter its localization or interaction with other proteins. For instance, PHR1 is SUMOylated by the E3‐SUMO ligase SIZ1 both in vitro and in vivo. PHR1 has been isolated with SUMO‐protein conjugates from Arabidopsis extracts, while *siz1* mutants showed upregulation of a group of *PSI* genes under P‐sufficient conditions. This evidence suggests a negative regulation of PHR1 by SIZ1 (Rojas‐Triana et al., 2013).

Protein phosphorylation is an important PTM‐regulating enzyme activity, protein localization and protein interactions. Changes in protein phosphorylation in response to Pi deficiency have been documented in plant roots (Li et al., 2014; Yang et al., 2019).

SnRK1 is a member of a family of protein kinases mediating cellular responses to different stresses that induce energy deficits. This protein kinase controls metabolic adaptation during low‐energy stress through transcriptional reprogramming by regulating the activity of several transcription factors (Baena‐González et al., 2007; Pedrotti et al., 2018; Tomé et al., 2014). The participation of SnRK1 in Pi deficiency signaling has been evaluated, and the kinase catalytic subunits were shown to be differentially regulated. When SnRK1α2 is degraded, SnRK1α1 is active. Plants *snrk1α1* growing on Pi‐deficient media exhibited modified transcription of specific genes and reduced starch degradation, suggesting an important role for this kinase in the response to such nutrient conditions (Fragoso et al., 2009). In this work, we extended the current knowledge of SnRK1 function during Pi starvation signaling. We observed that SnRK1 complexes, mainly formed with the SnRK1α1 catalytic subunit, are activated during −Pi stress. In silico predictions of the SnRK1α1 three‐dimensional structure and docking assays suggest feasible interaction modes of SnRK1α1 with peptides from PHR1, PHT:1;8, and PHO1. Recombinant complexes with SnRK1α1 activity were able to recognize and phosphorylate the peptides, particularly PHR1, which is the main transcription factor in the orchestration of gene expression during phosphate starvation. To elucidate the effect of this posttranslational modification on PHR1, we identified Ser11 as the main phosphorylation site and observed that S11A mutants did not show differences in their capability to bind DNA; however, transient transactivation assays in protoplasts indicated that phosphorylation of Ser11 in PHR1 acts as a negative regulator during Pi starvation.

## 2 MATERIALS AND METHODS

### 2.1 Plant materials and growth conditions

Wild‐type *Arabidopsis thaliana* ecotype Columbia (Col‐0) and *phr1* plants (SALK_067629.53.30.x) were grown in soil (sunshine 3, vermiculite, and agrolite in a proportion of 3:1:1) in a growth chamber at 22°C under short‐day conditions (8 h light/16 h dark). For the phosphate starvation treatment, seedlings were transferred to agrolite and watered every third day with Hoagland II solution containing either 500 μM ammonium phosphate (+Pi) as the only source of P or no phosphate (−Pi).

### 2.2 Identification of SnRK1 complexes in Pi‐deficient plants

Arabidopsis rosettes were harvested after 3 weeks of germination and frozen in liquid nitrogen. The leaves were ground, and total soluble protein was extracted in homogenization buffer containing 100 mM of tricine–NaOH (pH 8.0), 0.5 mM of ethylene glycol‐bis(2‐aminoethylether)‐N,N,N′‐tetraacetic acid (EGTA), 0.5 mM of ethylenediaminetetraacetic acid (EDTA), and 1 mM of benzamidine. Prior to homogenization, 1 mM of phenylmethylsulfonyl fluoride (PMSF), 1× protease inhibitor cocktail (Sigma), 1× phosphatase inhibitors (50 mM of NaF, 25 mM of β‐glycerolphosphate, 10 mM of sodium pyrophosphate, and 2 mM of sodium orthovanadate) and insoluble polyvinylpyrrolidone (2%, w/v) were added. The homogenate was centrifuged for 30 min at 13,000*g*, and insoluble material was removed. Crude extract (CE) was precipitated with 50% ammonium sulfate. The protein pellet was resuspended in SnRK1 buffer containing tricine–NaOH (pH 8.0), 0.5 mM EGTA, 0.5 mM EDTA, 1 mM benzamidine, 1 mM PMSF, 1× protease inhibitor cocktail (Sigma), and 1× phosphatase inhibitors (50 mM NaF, 25 mM β‐glycerophosphate, 10 mM sodium pyrophosphate, and 2 mM sodium orthovanadate). SnRK1 complexes from Pi‐deficient and Pi‐sufficient plants were compared using S‐300 sephacryl fractionation (HiPrep 16/60; GE Healthcare). Molecular weight markers were used to calibrate the column (Sigma).

### 2.3 SnRK1 activity assays

The SnRK1 activity assays were performed in a final volume of 25 μl. The reaction mixture was prepared with 40 mM HEPES (pH 7.5), 5 mM MgCl_2_, 200 μM ATP containing 12.5 kBq (γ‐^32^P) ATP (Perkin Elmer), 4 mM DTT, 1× phosphatase inhibitor, 1× protease inhibitor cocktail (Sigma), 200 μM AMARA peptide (AMARAASAAALARRR), PHT1;8 peptide (DMQRVMSRSHISRRR), PHR1 peptide (PVHRSGSRDLTRRR), PHO1 peptide (GLIKTYSSINMRRR), and NP‐GAPDH peptide (PVLRINSVEEGIRRR). The assay was started by addition of the sample containing protein kinase complexes. After 6 min, 15 μl of the reaction mixture was transferred to a square (2 × 2 cm) piece of phosphocellulose paper (Whatman P81), which was then immersed in 1% (v/v) phosphoric acid. The papers were rinsed twice with phosphoric acid, followed by acetone. The membranes were air‐dried, and the incorporation of ^32^P was quantified by the addition of scintillation liquid (ACSII aqueous counting scintillant, Amersham) and measurement in a Beckman scintillation counter.

### 2.4 Recombinant proteins

The cDNAs corresponding to SnRK1α1, SnRK1β1, SnRK1β2, SnRK1β3, SnRK1βγ, and SnAK2 were expressed as described by Maya‐Bernal et al. (2017). The kinase domain (KD) from SnRK1α1, SnRK1α2 and the activated kinase SnAK2 were cloned into the pGEX4T2 vector (GE Healthcare). For the γ‐^32^P incorporation assays, the KD from SnRK1α1 and SnRK1α2 were cloned into pMAL‐c vector (NEB). Full‐length PHR1 (At4G28610) and the nonphosphorylated mutant S11A were cloned into the pET28b(+) vector (Novagen). Point mutations were introduced according to the protocol described by Heckman and Pease (2007).

### 2.5 Phosphorylation assays

Both SnRK1α1 (KD) and SnRK1α2 (KD) proteins were previously activated in vitro by SnAK2. Protein activation was carried out in 25 μl using kinase buffer containing 40 mM HEPES‐KOH pH 7.5, 5 mM MgCl_2_, 200 μM ATP, 4 mM DTT, 1× phosphatase inhibitor (50 mM NaF, 25 mM β‐glycerol phosphate, 10 mM sodium pyrophosphate, 2 mM sodium orthovanadate) and 1× protease inhibitor cocktail (Sigma). The activation reaction was performed for 1 h at 30°C using 2 μg of SnRK1α (KD) and 0.2 μg of SnAK2.

Phosphorylation assays were performed using 0.5 μg of PHR1 and 1 μg of previously activated kinase domain SnRK1α1 (KD) or SnRK1α2 (KD). The reaction mixture was prepared with kinase buffer containing 2 μCi (γ‐^32^P) ATP (Perkin Elmer) and incubated for 1 h at 30°C, and the reaction was terminated by adding SDS‐Laemmli sample buffer. The phosphorylated proteins were separated by SDS–PAGE and transferred to an Immobilon‐P membrane (Millipore). Phosphorylation of the proteins was evaluated according to the incorporation of ^32^P, which was assayed using a Phosphoimager FX (Bio‐Rad).

### 2.6 Phos‐tag and western blotting

Phos‐tag electrophoresis was performed according to the manufacturer's instructions. Proteins were separated on SDS–PAGE gels containing 8% SDS, 25 mM Phos‐tag (WAKO, USA) and 50 mM MnCl_2_ at 15 mA for 2 h. After separation, the gels were incubated in transfer buffer containing 1 mM EDTA, followed by washing with transfer buffer without EDTA. Western blotting was performed by wet transfer onto PVDF membranes (Millipore), and signal detection was performed with an ECL kit (Millipore) according to the manufacturer's instructions. Antibodies against the HIS‐tag (Santa Cruz) were used to identify PHR1. Antibodies against all SnRK1 subunits were obtained as described in Maya‐Bernal et al. (2017).

### 2.7 Protoplast isolation

Arabidopsis protoplasts were isolated according to Wu et al. (2009) with some modifications. Peeled leaves adhering to the tape were transferred to a petri dish containing 10 ml of enzyme solution (20 mM MES pH 5.7, 1.5% [w/v] cellulase R10, 0.4% [w/v] macerozyme R10, 0.4 M mannitol and 20 mM KC, 10 mM CaCl_2_ and 0.1% BSA). The leaves were incubated in enzyme solution in the dark with gentle shaking for 2 h. Washing, dilution and transfection protocols were performed according to Yoo et al. (2007).

### 2.8 Protoplast transient reporter assays

The plasmids used for protoplast transient expression were a gift from Jan Lohmann (Addgene kit# 1000000036). A reporter vector (*pIPS::GUS*) was constructed using a 1 kb *IPS1* promoter region (Bustos et al., 2010) fused to the *GUS* gene and the *E9* terminator. The *PHR1* effector vector was constructed using the backbone of the pBRN168 plasmid (Addgene 79668), and the *DsRed* fragment was replaced with the *PHR1* or *PHR1 S11A* mutant. Protoplast assays were performed using *35 S*::*LUCIFERASE* as a transfection control vector at an effector:reporter:control ratio of 2:1:1. Nontransfected protoplasts (NT) were included as a negative control. GUS and luciferase assays were performed according to Yoo et al. (2007) using a CLARIOstar microplate reader (BMG Labtech). Relative GUS activity was reported as the ratio GUS/LUC. The values are given as the mean of at least two biological replicates with three technical replicates each.

mCherry‐PHR1/S11A fusion vectors were generated using the GreenGate cloning system (Addgene kit# 1000000036). Protoplast transfection was carried out using 15 μg of DNA.

### 2.9 Protein–protein interactions

To determine whether PHR1 interacts with SnRK1 catalytic subunits, pull‐down assays were carried out. GST‐SnRK1α1/α2 recombinant protein was incubated with PHR1‐HIS in an interaction buffer (IB; 20 mM Tris–HCl pH 7.5, 50 mM KCl, 5 mM EDTA, 5 mM MgCl_2_, 1 mM DTT, 2.5% [v/v] glycerol and 0.05% (v/v) NP‐40) for 2 h at 25°C with gentle agitation. After incubation, 30 μl of GSH‐agarose resin (Glutathione HiCap Matrix, QIAGEN) that had previously been equilibrated with IB buffer was added and incubated for 1 h at 25°C with gentle agitation. The resin was pelleted by centrifugation, and the supernatant was recovered (unbound fraction, UB). The pellet was washed three times with IB buffer, and the proteins were eluted using IB buffer containing 20 mM GSH. The same protocol was performed with GST protein as a control. The same amounts of different fractions were loaded on and separated by SDS–PAGE gels. The separated proteins were transferred to PVDF membranes, and the presence or absence of PHR1 was detected using anti‐HIS antibodies.

### 2.10 Electrophoretic mobility shift assay

The sense 5′ CAATTTTGGTAACG**GCATATTC**CATCGGATGATCCAAAATTCTCAAAA 3′ and antisense 5′ TTTTGAGAATTTTGGATCATCCGATG**GAATATGC**CGTTACCAAAATTG 3′ oligonucleotides, which correspond to the P1BS element (GNATATNC) in the *AtIPS1* gene, were used for electrophoretic mobility shift assay (EMSA) (Bustos et al., 2010). The sense oligonucleotide (750 nM) was ^32^P labeled using 5U T4 polynucleotide kinase (PNK) and 37 kBq [**γ**‐^32^P] ATP in a 10 μl reaction. The reaction was stopped at 67°C for 10 min, and the unincorporated label was discarded by passage through a G‐25 Sephadex column. Oligonucleotide annealing was performed using 20 nM labeled probe and 40 nM unlabeled antisense oligonucleotide in annealing buffer containing 10 mM Tris–HCl pH 8.0, 100 mM NaCl and 1 mM EDTA in a final volume of 60 μl. The reaction mix was heated at 94°C for 5 min and then cooled to room temperature for 2 h.

For EMSA, 0.5 μg of protein (unmodified or previously phosphorylated) was incubated with the annealed probe in a final volume of 30 μl with EMSA buffer (10 mM Tris–HCl pH 8.0, 100 mM NaCl, 10% [v/v] glycerol, 2 mM DTT and 0.05% [v/v] NP‐40). The mixture was incubated at 22°C for 30 min, and the reaction was stopped by adding 10× EMSA running buffer (10 mM Tris–HCl pH 8.0, 1 mM EDTA, 50% [v/v] glycerol, 0.001% [w/v] bromophenol blue and 0.001% xylene cyanol). Separation of the protein–DNA complex was carried out in a 4.5% acrylamide gel with 0.5× TBE buffer. After running, the gel was dried in a MODEL 543 gel drier (Bio‐Rad) and put into contact with an Imaging Screen HD (Bio‐Rad) for 24 h. Images were obtained using a Molecular Imager FX (Bio‐Rad).

### 2.11 Bimolecular fluorescence complementation assays in Arabidopsis roots

Protein–protein interactions were evaluated using bimolecular fluorescence complementation (BiFC) assays by cloning the genes *AtPHR1*, *AtSnRK1α1*, and *AtSnRK1α2* into the pSPYNE‐*35 S_GW* and pSPYCE‐*35 S_GW* vectors, which contain the N‐ and C‐termini of YFP, respectively. The cloning methodology used to generate these plasmids was based on GATEWAY technology (Anand et al., 2007; Walter et al., 2004). To produce these plasmids, the gene‐coding regions without stop codons were first cloned into the pENTR/D‐TOPO plasmid (Invitrogen) following the manufacturer's instructions to generate the entry constructs pENTR:*AtPHR1*, pENTR:*AtSnRK1α1*, and pENTR:*AtSnRK1α2*. Inserts were generated by PCR with the primers 5′*CACC*ATGGAGGCTCGTCCAGTTC3′ and 5′ATTATCGATTTTGGGACGCTTTGGC3′ for *AtPHR1*, 5′CACCATGGATGGATCAGGCACA3′ and 5′‐GAGGACTCGGAGCTGAGCAAG3′ for *AtSnRK1α1*, and 5′CACCATGGATCATTCATCAAAT3′ and 5′GATCACACGAAGCTCTGTAAG3′ for *AtSnRK1α2*. Subsequently, the entry constructs were digested with *Mlu*I, and the inserts containing the genes of interest were subjected to recombination procedures for transfer into the pSYNE‐*35 S_GW* and pSPYCE‐*35 S_GW* binary vectors using the LR‐clonase enzyme mix (Invitrogen) following the manufacturer's instructions. GFP fusion constructs were used as expression controls and were generated by cloning each gene into pEarleyGateway103 (pEG103; Earley et al., 2006) using the same procedure as for the BiFC vectors.

*Agrobacterium tumefaciens* PGV2260/C58 cells were transformed with each of the constructs generated in the binary vectors BiFC and pEG103. For BiFC assays, *Arabidopsis* roots were coincubated with the indicated combinations of transformant cells containing the constructs within the pSPYNE‐*35 S_GW* and pSPYCE‐*35 S_GW* vectors. For GFP expression, the roots were incubated only with *A. tumefaciens* containing the pEG103 construct.

*Arabidopsis* seedlings were transformed by cocultivation with *A. tumefaciens* as previously described (Campanoni et al., 2007; Grefen et al., 2010; Honsbein et al., 2009) with a few modifications. *A. tumefaciens* were grown until they reached the mid‐log phase of growth, and then the cells were harvested and incubated in sterile 10 mM MgCl_2_ plus 0.1 mM acetosyringone for 1 h at room temperature with gentle agitation. Then, the cells were harvested and resuspended to an OD_600_ of 0.5 in 0.5× Murashige and Skoog medium, pH 5.4, supplemented with 0.003% Sylwet L‐77. This final cell suspension was used to inoculate 3‐day‐old *Arabidopsis* seedlings and then coincubated under dark conditions for 24–48 h for GFP expression analysis or 72 h for BiFC assays.

### 2.12 Model of the three‐dimensional structure of SnRK1α1

A reliable prediction for the three‐dimensional structure of the SNRK1α1 subunit encoded by AT3G01090 was downloaded from the AlphaFold 2.0 (Jumper et al., 2021; Varadi et al., 2021) predictions database (alphafold.ebi.ac.uk/entry/Q38997). The overall confidence in the prediction was rated as very high for residues 17–279 and 290–344, 389–442 and 466–500 (Figure S1A). An independent evaluation of the appropriateness of the prediction was obtained with the ROSETTA design–HMMER approach (Martínez‐Castilla & Rodríguez‐Sotres, 2010; Rd.HMM). This protocol was able to correctly identify SNRK1α1 (UniProtKB accession Q38997) as the most appropriate sequence for the given backbone coordinates (after removal of sequence information) with a global score of 230.1 and *E*‐value of 2.1 × 10^−65^, while other sequences retrieved with statistically significant *E*‐values were all protein kinases but had considerably lower scores. The protocol score was divided into 2 domains. The first spanned residues 15–333 with an Rd·HMM score to sequence length ratio (Rd·HMMi) of 0.663 and an *E*‐value of 1.3 × 10^−66^, and the second domain spanned residues 394–508 with an Rd·HMMi of 0.15 and an *E*‐value of 8 × 10^−8^. Both scores rated the prediction as very good, especially for the catalytic core region 17–333, because the Rd·HMMi score was as good as the average value for X‐ray crystallography structures (Varadi et al., 2021). Among the list of proteins with folding patterns similar to SnRK1α1, judging from their Rd·HMM scores, we found several AURORA kinases from animal species. The segment from residues 41 to 296 of human AURORA‐A kinase (Marumoto et al., 2002) was found to fit the folding from positions 17–274 of the probabilistic hidden Markov model, with an Rd·HMMi of 0.390 and an *E*‐value of 2.3 × 10^−29^. Superposition of the catalytic cores of the SnRK1α1 AlphaFold 2.0 model with the X‐ray structure of human AURORA‐A kinase (Figure S1B) shows excellent agreement with the alignment produced by Rd·HMM. With this last kinase as a reference, it was possible to dock the substrate ATP or the product ADP and two essential Mg^2+^ atoms, as well as to predict an appropriate conformation for the phosphorylated residue Thr175, which is not considered by AlphaFold 2.0. The potentially phosphorylated residue Ser176 has no equivalent in AURORA‐A kinase and was added manually.

The geometry was made consistent with quantum mechanics (QM) using geometry optimization under the semi‐empirical PM7 level of theory with localized molecular orbitals (LMO‐PM7) and implicit solvation with COSMO, as implemented in MOPAC 2016 (Stewart, 2016). Optimization was performed with the AlphaFold 2.0 prediction as a geometric reference to keep the conformation reasonably close to the original prediction.

### 2.13 Molecular docking of phosphopeptides

Phosphopeptide docking was performed using AutoDock Vina 1.2.3 (AdVi; Eberhardt et al., 2021). This software has been extensively used to explore the possible docking poses of diverse ligands in macromolecules, mainly in proteins. However, its use in the docking of peptides is limited due to the large number of rotating bonds. The most recent version of this software features several improvements, and we found it feasible to explore the docking of molecules with up to 81 rotating bonds. Phosphorylated versions of the peptides were built using Chimera (Pettersen et al., 2004) and prepared for AdVi using Open Babel (O'Boyle et al., 2011).

The receptor was set as the predicted structure of SnRK1α1 with ADP and two Mg^2+^ atoms with the docking box enclosing the full binding pocket and neighboring groups. This strategy aims at the formation of the enzyme–product complex in silico, and it was found to be a convenient way to guide the docking of the residue known to be phosphorylated without introducing empirical constraints.

Several docking rounds were performed with each peptide, and the results were analyzed in terms of the position of the phosphorylated residue and the orientation of the N‐ and C‐termini. Specifically, since the peptide is only part of a protein, its termini should not be buried.

Those conformations meeting the above criteria were subjected to further geometry optimization with MOPAC 2016, as described for the preparation of the model in the preceding section. For selected conformations, the binding energy was estimated from the PM7‐LMO enthalpy of formation of the complex minus the PM7‐LMO enthalpy of formation of the dissociated species (protein and peptide). This method allows a better evaluation of the interaction energy because it includes electronic effects, which are neglected in classical molecular force fields and help to better identify strong complexes.

### 2.14 Statistical analysis

The experimental data were analyzed using GraphPad Prism 9 software and are shown as the mean ± sd. Comparisons between different groups were performed by one‐way and two‐way anova. A *p‐*value of less than 0.001 was considered statistically significant.

### 2.15 Accession numbers

Sequence data in this article can be found in The Arabidopsis Information Resource (TAIR) under the following accession numbers: AT3G01090 (SnRK1α1), AT3G29160 (SnRK1α2), AT5G21170 (SnRK1β1), AT4G16360 (SnRK1β2), AT2G28060 (SnRK1β3), AT1G09020 (SnRK1βγ), AT3G45240 (SnAK2), and At4G28610 (PHR1).

## 3 RESULTS

### 3.1 Presence of active high‐molecular‐weight complexes during Pi starvation

**FIGURE 1:**
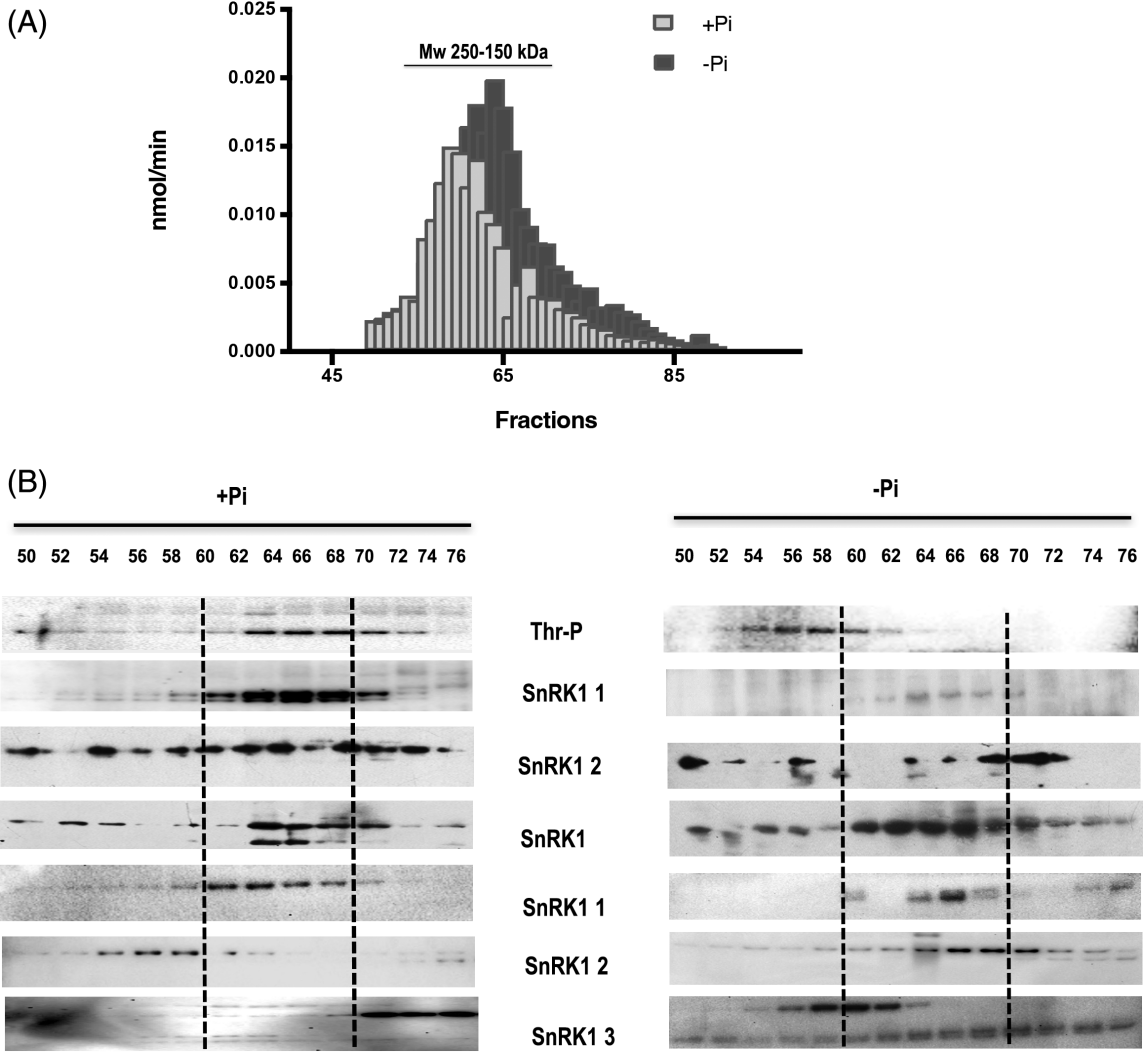
Gel filtration on a Sephadex S 300 column of SnRK1 activity in protein extracts from plants grown under Pi‐sufficient (+Pi) or Pi‐deficient conditions (−Pi). (A) SnRK1 activity in different fractions of high‐molecular‐weight complexes. The column was calibrated using molecular markers, and the activity peak corresponded to masses between 250 and 150 kDa. (B) Western blot of fractions in (A) developed with antibodies against different SnRK1 subunits or against the phosphopeptide (pThr) shared by the active forms of SnRK1α1 and SnRK1α2. The vertical dotted lines indicate the fractions inside the activity peak

SnRK1 is a protein kinase responsible for some of the responses to cellular energy deficit. It promotes the phosphorylation of several proteins (e.g., enzymes, transcription factors, transporters) to modify metabolic processes and gene expression. During nutrient starvation, particularly long periods of phosphate deficiency, ATP and ADP levels are reduced, which promotes metabolic modifications. Under this condition, the SnRK1α2 subunit is differentially regulated and targeted for degradation, while SnRK1α1 retains high activity (Fragoso et al., 2009). To identify the subunits forming part of the active complex during Pi starvation, we partially purified high‐molecular‐weight complexes with SnRK1 activity. We observed that the activity increased during Pi starvation (Figure 1A), and high‐molecular‐weight complexes (between 250 and 150 kDa) were formed with SnRK1α1 as the main catalytic subunit and SnRK1β1, SnRK1β2, SnRK1β3 and SnRK1βγ as regulatory subunits (Figure 1B). In comparison, complexes formed under Pi‐sufficient conditions contained both catalytic subunits, but the regulatory subunits SnRK1β2 and SnRK1β3 were not included in the complex. The low activity in low‐molecular‐weight fractions (beyond fraction 70) is difficult to ascribe to a particular SnRK1 catalytic subunit, which could be due to SnRK2 enzymes that are able to phosphorylate the AMARA peptide.

### 3.2 Phosphorylation of PHR1

**FIGURE 2:**
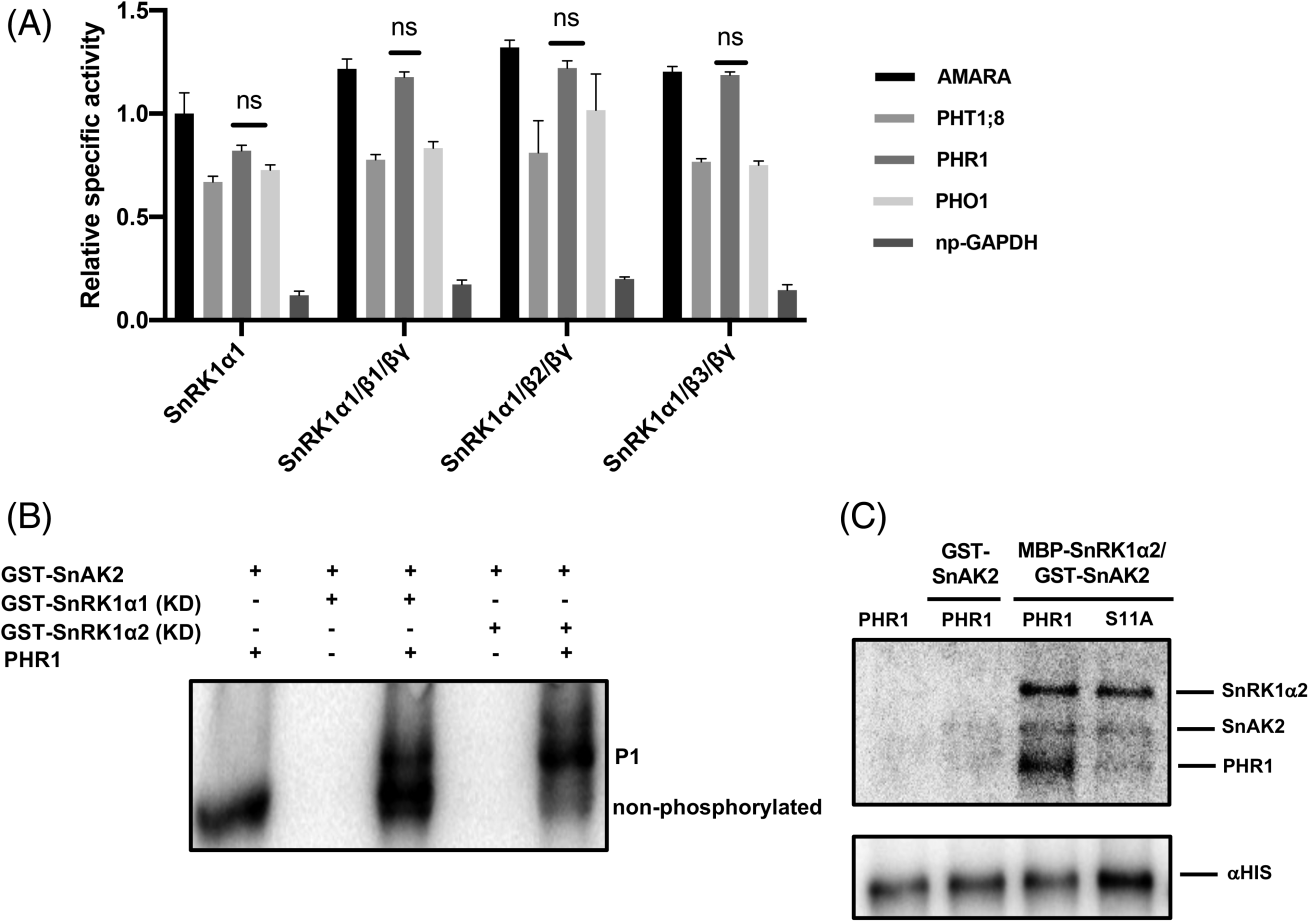
PSR proteins as SnRK1 substrates. (A) SnRK1 activity on PHT1;8, PHR1, PHO1 and NP‐GAPDH peptides. Specific activity was expressed relative to the activity of SnRKα1 and the AMARA peptide as substrate (set to 1). The results are the mean ± sd from three different experiments. Statistical analysis was performed using two‐way anova. The comparison between the different peptides was made for each SnRK1 complex. In all cases, the activity on AMARA and PHR1 did not show significant differences (ns). The other peptides showed differences from the AMARA peptide (****; *p* < 0.0001). (B) Phos‐tagged gels showing the phosphorylation status of PHR1 after incubation with SnRKα1 and SnRKα2. SnRK1 activated kinase (SnAK2) was included as a control. (C) Incorporation of ^32^P‐γATP into PHR1 catalyzed by SnRK1. Proteins were separated by SDS–PAGE and transferred to PVDF membranes. Incorporation of ^32^P was detected using an imaging screen and the Phosphoimager FX (Bio‐Rad). PHR1 was detected with an anti‐HIS antibody to demonstrate the correct loading in all lanes

Several types of proteins have been identified to participate in Pi deficiency signaling and adaptation. To determine whether SnRK1 could phosphorylate some of these proteins, we performed a computer search using ScanProsite to identify the presence of the SnRK1 target motif (Table 1A). Selected proteins identified to have at least one phosphorylated motif included a member of the PHT1 family of phosphate transporters (PHT1;8), a phosphate transporter involved in the transfer of Pi from root epidermal and cortical cells to the xylem (PHO1), the main transcription factor that regulates the expression of Pi‐deficient genes (PHR1) and a metabolic enzyme involved in a glycolytic bypass reaction during Pi deficiency (NP‐G3PD, Table 1B; Plaxton & Tran, 2011). Peptides of 15 aa were synthesized around the putative phosphorylated residue and were used as substrates in SnRK1 activity assays. The SnRK1α1 catalytic subunit alone and with different βγ/β dimers was used to generate different kinase complexes. As reported previously (Maya‐Bernal et al., 2017), SnRK1 activity was higher when the catalytic subunits were associated with two of the regulatory subunits using the AMARA peptide. Interestingly, different SnRK1 complexes could recognize and phosphorylate peptides from PHR1 at almost the same level as the AMARA peptide (Figure 2A). Surprisingly, the NP‐GAPDH peptide was phosphorylated at only low levels by SnRK1 complexes although this protein was described as a target of these kinases (Piattoni et al., 2011). Phosphorylation of PHR1 recombinant protein was observed with both SnRK1α1 and SnRK1α2 catalytic subunits, but the in vitro activity of recombinant protein containing SnRK1α2 was much higher; for this reason, all of the subsequent in vitro phosphorylation assays in this work were performed with SnRK1α2 (Figure 2B). Phosphorylation of PHR1 indicated in PhosPhAt, a database of phosphorylation sites in Arabidopsis proteins, showed S11 residues phosphorylated in in vivo experiments (Durek et al., 2010), and according to the results obtained using ScanProsite, Ser11 is contained inside a SnRK1 consensus motif (Table 1C). To determine whether SnRK1 could phosphorylate the S11 residue in PHR1, ^32^P assays were carried out using recombinant proteins. The results showed that PHR1 is phosphorylated by SnRK1 and that mutation of S11 by alanine dramatically reduced the signal. This result strongly suggested that this is the major recognition site for SnRK1 (Figure 2C).

**TABLE 1:**
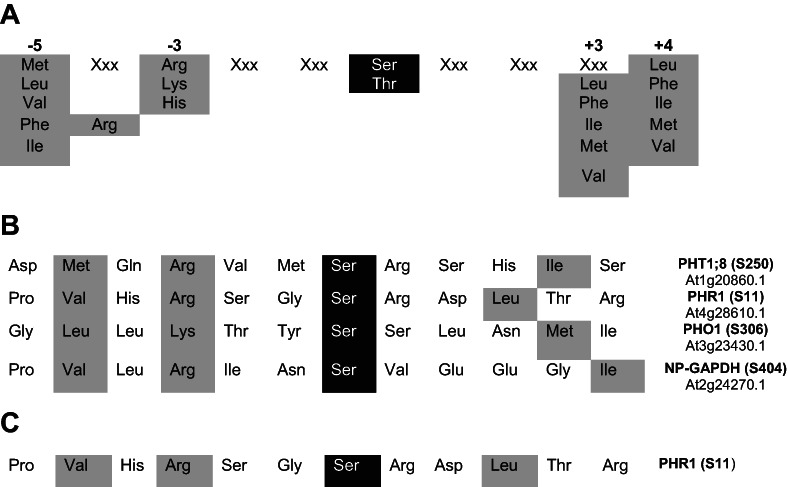
(A) Motif recognized by the SnRK1 family of protein kinases. In black, the phosphorylated amino acid and in gray the conserved amino acids around the phosphorylated residue. (B) ScanProsite search for proteins involved in phosphate starvation responses (PSRs) that contain at least one SnRK1 motif. (C) Identification of one SnRK1 motif in PHR1.

### 3.3 Model of the three‐dimensional structure of the SnRK1α subunit and docking with PSR protein phosphopeptides

**FIGURE 3:**
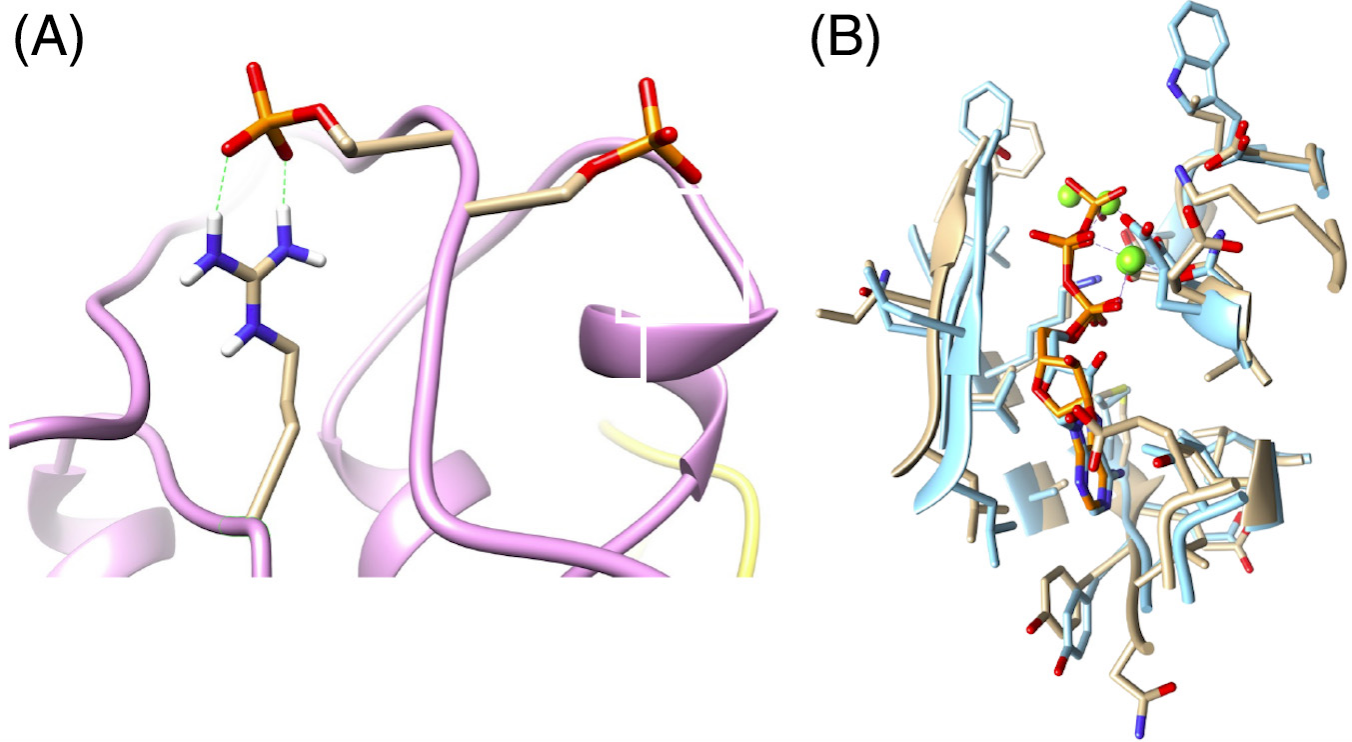
Details of the three‐dimensional structure of SnRKα1 predicted by AlphaFold 2.0 (alphafold.ebi.ac.uk/entry/Q38997). (A) Magnified view of the two loop connectors within the central domain of SnRKα1 (enclosed by a black square in Figure S1B) and showing the anchor between Arg141 and phospho‐Thr155. (B) Magnified view of the cleft between domains 1 and 2 of the predicted SnRKα1 structure compared to the corresponding site in AURORA‐A kinase (corresponding to the zone enclosed by a black square in Figure S1C). In (B), ADP and ATP are shown as licorice structures, and Mg^2+^ atoms, two for each structure, are shown as green spheres. The images were prepared using Chimera (Pettersen et al., 2004). Some hydrogen atoms were omitted for clarity

Due to the evidence suggesting that SnRK1 phosphorylates some proteins mediating the response to phosphate deficiency, three‐dimensional models were generated to determine the feasibility of the interaction between the SnRK1 catalytic subunit and these proteins. The three‐dimensional structure of any SnRK1 protein from Arabidopsis has not yet been solved, but it was predicted by AlphaFold 2.0. The prediction seems to be a very good approximation to the true native structure of SnRK1α1, especially for the catalytic core, judging by the confidence score and the Rd·HMMi of the backbone (Figure S1A). SnRK1α1 features 4 domains, which are presented in the figure as colored cartoon structures. Domain 1, at the N‐terminus, is a compact αβ domain (cyan). Domain 2 is a central α domain with close contacts with domain 1 (magenta). At the interface between domains 1 and 2, there is a cleft where the putative active site is located (see below). Domain 3 forms a sort of cover lying under domains 1 and 2 (yellow). Finally, domain 4 has a long intrinsically disordered linker and a small α + β C‐terminal segment and seems to have little interaction with the rest of the protein (red, Figure S1B). The phosphorylated residues Thr175 and Ser176 are positioned in one of the two long loops connecting the α‐helix segments in domain 2 (Figure 3A; Figure S1B boxed zone). Phospho‐Thr175 seems to have a strong interaction with Arg141, which is located at a neighboring shorter loop.

The comparison of the predicted active site with the active site of the cell cycle regulator AURORA‐A kinase (Marumoto et al., 2002) revealed notable conservation in both the folding pattern (Figure S2C) and the orientation of the side chains (Figure 3B; Figure S1C boxed zone). Since AlphaFold 2.0 may use templates but does not depend on them, the coincident folding is unlikely to be an artifact from the threading of the target sequence to a template; in fact, the AlphaFold 2.0 EvoFormer block does not perform any kind of threading or template superposition (Jumper et al., 2021). Currently, AlphaFold 2.0 cannot predict the structures of phosphorylated proteins, but by taking advantage of the significant similarity between these two proteins, we were able to predict the conformation of the phosphorylated Thr175 in contact with the guanidino group of Arg141. There was no need for any change in the backbone, and this contact should add stability to domain 2; thus, these two proteins might share their mechanism of activation by phosphorylation. These phosphorylated residues are located in one corner of the active site cleft and may be important for the regulation of kinase specificity.

**FIGURE 4:**
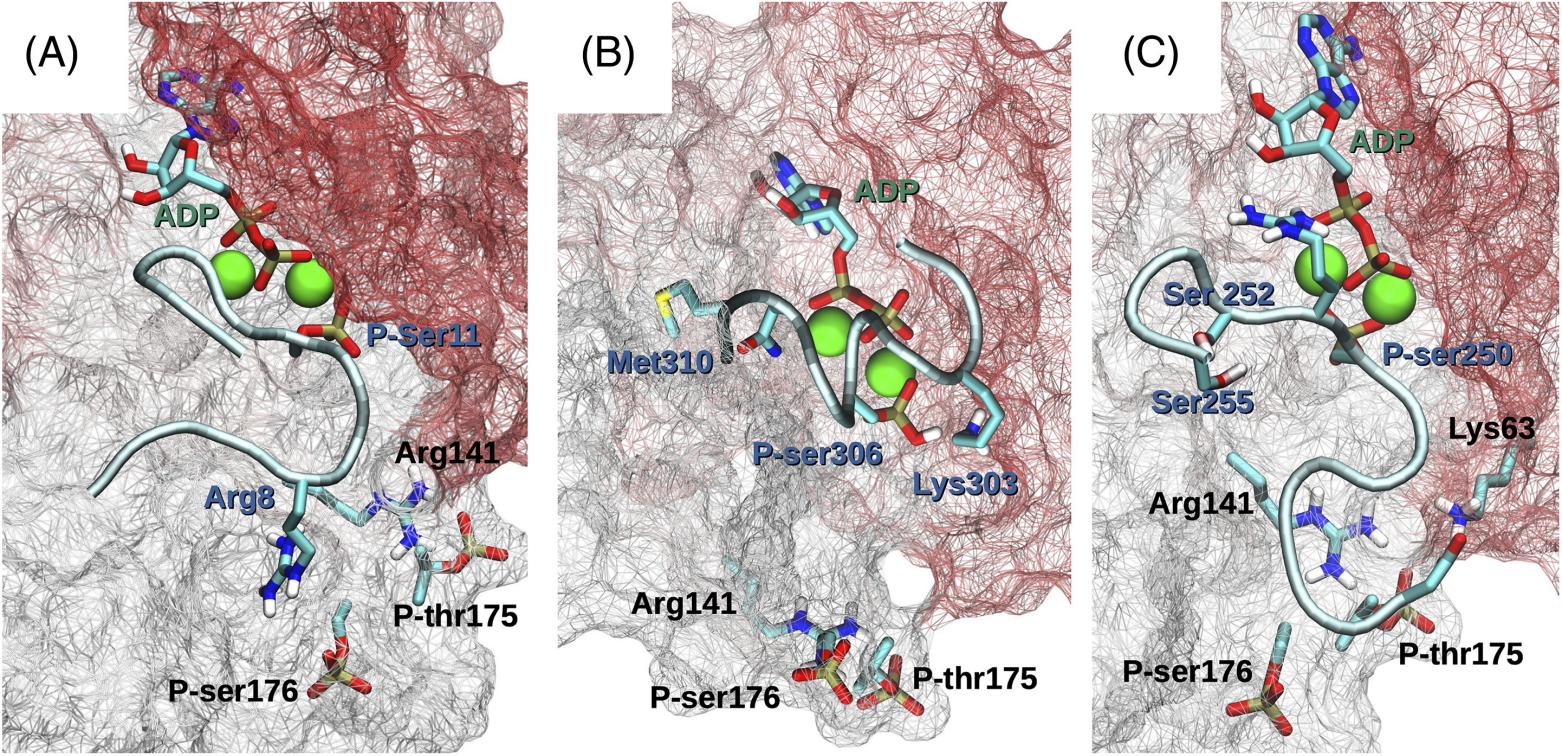
Top‐ranked docking poses for three phospho‐peptides in complex with the SnRK1α1‐ADP‐Mg^2^‐predicted three‐dimensional structure. The protein residues within 8.5 Å from the peptide are shown as a surface mesh (color is arbitrary); the peptide backbone is shown as a tube in metallic light blue; ADP and some selected residues are shown as licorice models, colored by element; and Mg^2+^ is shown as green spheres. Labels in black correspond to the enzyme, labels in blue are residues of the product peptide, and ADP is in green. (A) AtPHR1|5–16|Pser11. (B) AtPHO1|301–316|Pser306. (C) AtPHT1;8|244–255|Pser250. The molecular graphics were prepared with VMD (O'Boyle et al., 2011). The orientation of the view differs in the three panels, and only selected polar hydrogens are shown

The target peptide specificity of protein kinases is one of the areas in which predictions are unreliable, and, currently, the number of enzyme–substrate complexes experimentally solved is relatively low. Relaying on the high quality and appropriateness of the structural prediction for the SnRK1α catalytic core, we attempted to dock SnRK1α with the three different peptides from PHT1;8, PHO1 and PHR1, which are known to be targets of this kinase (Figure 2A). A large number of results are generated during protein–protein docking, and the target peptides contain more than one serine or threonine in their sequence, complicating the analysis and classification of docking results. To address this problem, we tried to predict not the enzyme substrates but the enzyme‐ADP‐Mg^2+^‐phosphopeptide (enzyme–products) complex. This tags the phosphorylation site and simplifies the analysis because the phosphate group in the product is expected to sit close to the ADP β‐phosphate and one of the Mg^2+^ atoms. The binding enthalpy of the complexes was then estimated using PM7‐LMO calculations, as described in the methods. Three enzyme–product complexes, SnRK1α1/PHR1, SnRK1α1/PHT1;8, and SnRK1α1/PHO1, are shown in Figure 4A–C. These candidate structures were chosen based on the geometry of the phospho‐amino acid in relation to the active site, the exposure of the N‐ and C‐termini (the pose should be compatible with the peptide segment as part of a longer protein), and the PM7‐LMO ∆H of formation of the complex (Table S1). These results strongly suggest how these three peptides from PSR proteins might dock within the catalytic domain of SnRK1α1 and make good candidates for specific substrates.

### 3.4 In vitro and in vivo interactions between SnRK1α subunits and PHR1

**FIGURE 5:**
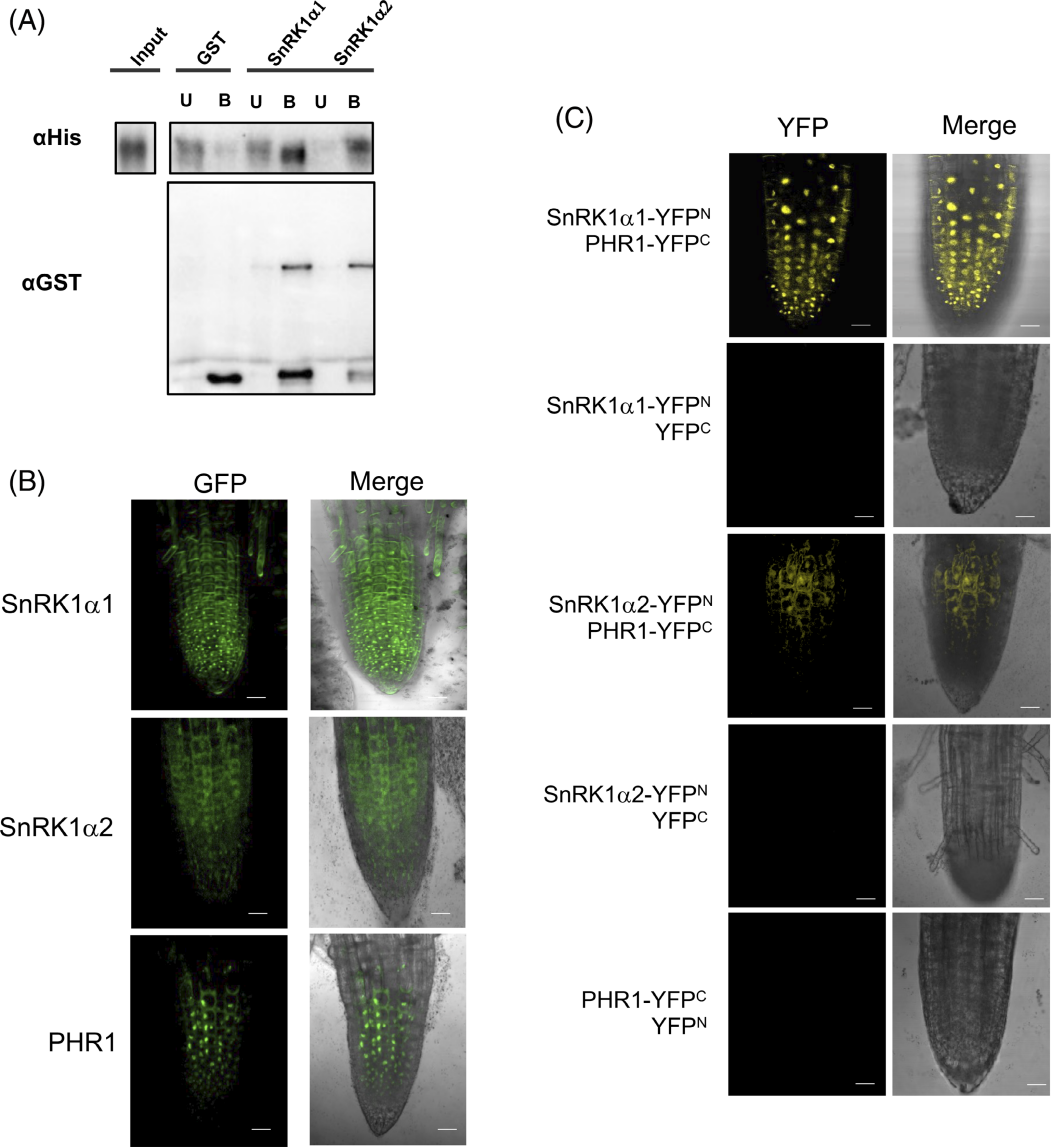
Interaction between SnRK1 and PHR1. (A) Pull‐down assays using recombinant proteins PHR1‐HIS and GST‐SnRK1. GST‐SnRK1 was bound to the glutathione‐agarose column. Unbound (U) and bound (B) PHR1 were identified using antibodies against HIS. SnRK1 was identified as associated with the column using anti‐GST antibodies. (B) Cytoplasmic and nuclear localization of SnRK1α1, SnRK1α2 and PHR1 fused to GFP in transient *Agrobacterium* root infections (see Section 2). (C) YFP complementation using SnRK1α1‐YFP^N^ and SnRK1α2‐YFP^N^ with PHR1‐YFP^C^. Negative controls consisting of SnRK1 α1‐YFP^N^/YFP^C^, SnRK1α2‐YFP^N^/YFP^C^, and PHR1‐YFP^C^/YFP^N^ were included. Scale bars: 20 μm

The recognition of PHR1 as a target of phosphorylation by SnRK1 and the interaction between the phosphopeptide and the catalytic subunit predicted by docking simulations suggest a direct interaction between these two proteins. To investigate this possibility, we performed pull‐down assays using GST‐SnRK1α1/α2 catalytic domains and PHR1‐His recombinant proteins. As shown in Figure 5A, PHR1 was found in the bound fraction (B), interacting with SnRK1α1 and SnRK1α2. As a negative control, GST failed to pull down any SnRK1.

To elucidate whether PHR1 and SnRK1 could interact in vivo, BiFC assays were performed by transient expression of their cDNAs using *Agrobacterium* in roots of Arabidopsis seedlings. Localization of the GFP fusion proteins showed that SnRK1 was present in the cytoplasm and nucleus, whereas PHR1 was mainly found in the nucleus in root cells (Figure 5B). The interaction between SnRK1 catalytic subunits and PHR1, as suggested by YFP complementation, occurred mainly in the nucleus for the SnRK1α1 subunit and in both the cytoplasm and nucleus for SnRK1α2. The lack of complementation with the empty vectors confirmed that this is a specific interaction (Figure 5C).

### 3.5 Effect of phosphorylation on PHR1 activity

**FIGURE 6:**
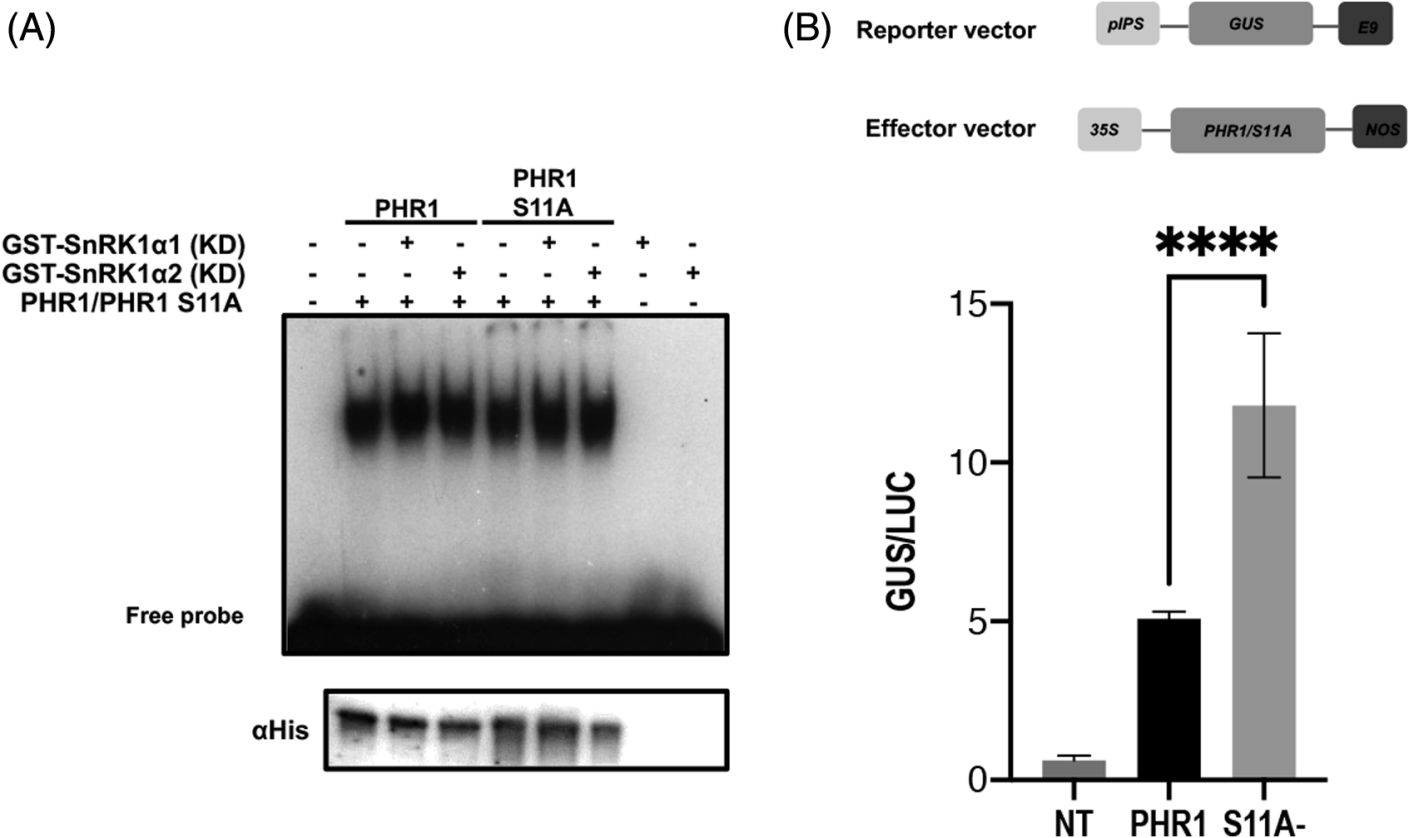
Effect of PHR1 phosphorylation. (A) The electrophoretic mobility shift assay using a probe containing one P1BS and PHR1‐HIS or S11A‐HIS mutant protein. The presence (+) or absence (−) of the catalytic domain of the recombinant fusion protein GST‐SnRK1α1 or GST‐SnRK1α2 is indicated. Corroboration of the presence of PHR1 in all lines was indicated using an anti‐HIS antibody. (B) Transient PHR1 activity was introduced into Arabidopsis protoplasts by transfection with a reporter vector carrying *pIPS*::*GUS* and effector vectors containing *p35S*::*PHR1* or *p35S*::*S11A*. The control vector carried *p35S::LUC*. NT represents a nontransfected protoplast. After transfection, the protoplast was incubated for 16 h. relative GUS activity was reported as the ratio GUS/LUC. The values given are the means of at least two biological replicates with three technical replicates. Statistical significance was determined via a one‐way ANOVA. Asterisks indicate significant differences (*p* < 0.05).

To determine the effect of PHR1 phosphorylation on the ability of PHR1 to bind DNA, mobility shift assays were carried out using the P1BS recognition motif. Both wild‐type PHR1 and its S11A mutants were able to bind P1BS with or without SnRK1 incubation, suggesting that this posttranslational modification did not interfere with DNA binding activity (Figure 6A). Additionally, PHR1 and S11A did not alter the protein stability since transient expression in Arabidopsis protoplasts showed the presence of the protein regardless of the phosphate availability (Figure S5). The transactivation activity of PHR1 was evaluated using an in vivo assay in Arabidopsis protoplasts. The reporter vector carried the promoter region of the *IPS* gene, which contains two P1BS motifs fused to the *GUS* gene. The effector vector carried the *PHR1* gene in its WT or S11A form under the regulation of the *35 S* promoter, and the normalized vector carried the *LUC* gene under the regulation of the *35 S* promoter. The relative GUS activity was measured in transfected protoplasts to assess the expression level of the reporter. GUS activity showed a significant increase in protoplasts transfected with the S11A mutant compared to WT version (Figure 6B), suggesting that phosphorylation of PHR1 at Ser11 acts as a negative regulator of its transactivation activity.

## 4 DISCUSSION

### 4.1 SnRK1 activity during Pi starvation

SnRK1 is a key protein kinase that regulates multiple processes in plant growth and development. It is involved in the responses to different types of abiotic stresses. We have documented that the catalytic subunit SnRK1α2 is degraded in response to Pi deficiency, and in turn, SnRK1α1 is involved in the regulation of carbohydrate metabolism, particularly starch degradation (Fragoso et al., 2009), under Pi deficiency. With the aim of extending our current knowledge on the function of SnRK1 during the Pi deficiency response, we evaluated its kinase activity in plants grown under Pi‐deficient and Pi‐sufficient conditions. SnRK1 activity was higher under Pi‐deficient conditions than under Pi‐sufficient conditions. The peak kinase activity coincided with a range of molecular masses between 250 and 150 kDa, suggesting the presence of multiprotein complexes. Evaluation of all the SnRK1 subunits in those fractions revealed different proportions of catalytic and regulatory subunits under the +Pi and –Pi conditions. In particular, the data suggest the presence of SnRK1α1/βγ/β2 and SnRK1α1/βγ/β3 complexes specifically under stress conditions. Consistent with observations of its animal and yeast homologs, SnRK1 has been recognized as a heterotrimeric enzyme formed by a catalytic alpha subunit and two regulatory subunits. However, some results also suggest the possibility that the catalytic subunits can act as complex‐independent kinases (Ramon et al., 2019), a mechanism completely different from what has been proposed in animals and yeast (Martínez‐Barajas & Coello, 2019). In this study, we observed basal kinase activity at molecular weights of approximately 60 kDa (fractions 72–76), but we detected only a slight amount of the activated SnRK1α2 subunit in the same fractions under normal growth conditions (+Pi). We assume that this basal activity is attributable to other kinases that also use the AMARA peptide as a substrate, such as SnRK2. These results support a role for differential SnRK1 protein complexes under both normal and stress conditions but are not consistent with a role for the isolated SnRK1α1 or SnRK1α2, at least under the conditions tested.

### 4.2 Pi deficiency proteins as substrates for SnRK1 phosphorylation

Pi starvation is a stress condition that promotes major changes at the cellular and whole‐plant levels. In Arabidopsis, the transcriptomic changes associated with Pi starvation have been analyzed, and the modified expression of thousands of genes was documented (Fang et al., 2009). A MYB‐CC family of transcription factors comprising PHR1 and PHR1‐like (PHL) proteins is involved in the regulation of gene expression in response to Pi starvation. In fact, it was demonstrated that PHR1 and any of the redundant PHL1 and PHL2 transcription factors are involved in the regulation of more than 80% of the genes whose expression is modified by Pi deficiency in wild‐type plants (Barragán‐Rosillo et al., 2021; Bustos et al., 2010). Despite being an important regulator of Pi deficiency responses, very little is known about the mechanism that drives PHR1 activation. Posttranslational modification (PTM) of PHR1, such as sumoylation by AtSIZ1, has been partially implicated in the positive control of *AtIPS1* and *AtRNS1* expression; however, it does not have any effect on the regulation of other genes involved in Pi deficiency adaptation (Miura et al., 2005). Protein phosphorylation is another PTM that is associated with responses to Pi deficiency in both rice and Arabidopsis. Proteins involved in different processes show changes in phosphorylation status in response to Pi deficiency or Pi resupply after deficiency (Mehta et al., 2021; Yang et al., 2019). We evaluated whether SnRK1 was able to recognize and phosphorylate some of the proteins associated with PSR, focusing on several proteins that contained the motif for SnRK1 phosphorylation. The analysis of phosphorylation by different complexes showed that the transcription factor PHR1 was recognized and phosphorylated at almost the same level as the canonical substrate AMARA. PHO1 and PHT1;8 were also phosphorylated, although to a lesser extent, by the different complexes. Intriguingly, the peptide associated with NP‐GAPDH was not recognized, despite being described as a substrate for SnRK1 partially purified from wheat endosperm. It is possible that the structure of the small peptide was not sufficient for interaction with SnRK1, or perhaps the SnRK1 from wheat endosperm included additional associated proteins that enhanced the interaction (Piattoni et al., 2011); more work is clearly needed to explore this discrepancy. Members of the PHT1 family of transporters in rice have been identified as substrates for CASEIN KINASE 2 (CK2), and PTMs negatively regulate their translocation to the plasma membrane (Yang et al., 2019). In Arabidopsis, PHO1 has been described as a phosphoprotein in PhosPhAt (Durek et al., 2010), with phosphorylation hotspots at Thr304 and Tyr305, but no data on Ser306 phosphorylation have been published. Moreover, the biological function of these changes has not been described.

Three‐dimensional models of the catalytic domain of SnRK1α1 in complex with phosphopeptides of PHR1, PHT1;8 or PHO1 are consistent with a stable interaction between these three protein targets. The docking conformations of the phospho‐amino acids are similar and expected since there is a favorable interaction between the phosphate group and one of the Mg^2+^ atoms. However, there is a distinct orientation of other residues in each of the three peptides. While the central portion of peptides AtPHR1|5–16|Pser11 (Figure 3A) and AtPHT1;8|244–255|Pser250 (Figure 3C) are in the extended conformation and lie parallel to the ADP product, the peptide AtPHO1|301–316|Pser306 (Figure 3B) is in a helicoidal conformation and lies almost transversal to the ADP longitudinal axis. The N‐terminal portions of peptides AtPHR1|5–16|Pser11 and AtPHT1;8|244–255|Pser250 form a similar hook stabilized by intramolecular interactions, but their C‐terminal sides form loops with different orientations. In addition, their stability depends on interactions with different residues in the enzyme. Despite their conformational differences, all three peptides share several interactions, for instance, Lys144 H‐bonds to the backbone carbonyl oxygen of Pser11 in AtPHR1|5–16|Pser11 (Figure S2, see K144, orange arrow), Pser306 in AtPHO1|301–316|Pser306 (Figure S3, see K144, orange arrow) and Ser252 in AtPHT1;8|244–255|Pser250 (Figure S4, see K144 orange arrow). Apparently, this residue is an important component of peptide binding in this enzyme. Some residues responsible for hydrophobic contacts also coincide in two or three of the complexes, such as Pro180 (Figures S2–S4, see P180, brown arrow).

Finally, to provide a quantitative estimate of the strength of these interactions, we determined the magnitude ∆H of formation (Table S1). The free energy of interaction between a protein and a ligand is indicative of the stability of the complex and has one enthalpic (∆*H*) and one entropic (T∆*S*) component. Here, the ∆*H* of formation gives an estimate of the enthalpic component, while the solvent‐accessible surface (∆SAS) is related to an increase in entropy due to the transfer of solvent from the protein surface to the bulk solvent, although other factors, harder to assess in silico, also contribute to this last component.

All three complexes of phosphopeptides with SnRK1α1‐ADP‐Mg^2+^ are predicted to have a negative ∆H of formation, and all these phosphopeptides are likely to bind to SnRK1 with good affinity. In agreement with LigPlot+ analysis, the SnRK1α1‐ADP‐Mg^2+^ complex with AtPHO1|301–316|Pser306 has fewer polar contacts, its binding is thus dominated by hydrophobic interactions, and it shows the smallest magnitude ∆H of formation (Table S1). In contrast to this complex, the complex of AtPHT1;8|244–255|Pser250 with SnRK1α1‐ADP‐Mg^2+^ has many polar and nonpolar contacts, and its ∆H of formation is the highest in magnitude (more negative). This last complex also produces the largest decrease in the solvent‐accessible surface (∆SAS). Unfortunately, enzyme specificity depends not only on equilibrium thermodynamics but also on kinetic factors that cannot be assessed in silico without detailed knowledge of the chemical mechanism of catalysis; therefore, the present estimates do not indicate which substrate would be phosphorylated with greater specificity by SnRK1α1.

### 4.3 SnRK1 phosphorylates the S11 residue in PHR1, inhibiting its transactivation activity

The interaction of PHR1 with SnRK1 was corroborated in vitro using recombinant proteins. In vivo BiFC experiments also showed an interaction between SnRK1α1 and SnRK1α2 with PHR1 in both the nucleus and the cytoplasm in root cells (Figure 4). PHR1 has been identified as a phosphoprotein through several phosphoproteome experiments investigating the response to biotic, abiotic or hormonal treatments (Durek et al., 2010). There is an important hotspot in the N‐terminal region corresponding to residues Ser11 through Ser32. In that region, Ser11 is part of a SnRK1 phosphorylation motif, VHRSG **S** RDL, where the underlined serine is the phosphorylated residue. Full‐length PHR1 was phosphorylated by the catalytic domains of both SnRK1 catalytic subunits. Separation of the phosphoproteins by Phos‐tag gels identified a phosphorylated band, and mutation of S11 seems to disrupt ^32^P incorporation, suggesting that S11 is a major phosphorylation site for SnRK1. The participation of other protein kinases is another factor that has not yet been considered, and we cannot rule out the possibility that different protein kinases phosphorylate PHR1 in response to Pi starvation, particularly in the hotspot defined between Ser11 and Ser32, but clearly additional experiments should be performed.

Phosphorylation can modify protein stability. It has been observed that overexpression of SnRK1α1 delays the degradation of FUSCA 3, although it is not known whether this effect is direct or indirect (Tsai & Gazzarrini, 2012).

DNA mobility shift assays using the P1BS binding motif did not support a change in the DNA binding ability of either phosphorylated or nonphosphorylated PHR1. This result is not surprising since the MYB DNA‐binding domain is distant from the phosphorylation site (Jiang et al., 2019). The transcriptional activity of PHR1 was evaluated by cotransformation of a reporter vector carrying the *IPS1* promoter fused to the *GUS* gene and a wild‐type or S11A version of PHR1 in *Arabidopsis* protoplasts. Notably, incubation of protoplasts showed that wild‐type PHR1 had less activity than the S11A mutant, suggesting that phosphorylation of Ser11 in PHR1 negatively regulates its activity on the *IPS1* promoter in a transient expression system in Arabidopsis protoplasts.

Differences in transcriptional activity found between PHR1 and S11A might be due to changes in protein stability. Transient expression of S11A and PHR1 in protoplasts did not show important differences (Figure S5). However, Navarro et al. (2021) showed that plants growing in Pi deficiency had higher amounts of PHR1 when they were pretreated with the proteosome inhibitor MG132, suggesting the participation of the ubiquitination machinery in PHR1 stability. Some results have indicated that phosphorylation might be coupled to the proteasome to control the stability or activity of transcriptional regulators. Zhai et al. (2017) demonstrated that phosphorylation of the transcription factor WRINKLED1 (WRI1) by SnRK1α1 rapidly induces its degradation. PHR1 phosphorylation might stimulate its degradation via the proteasome as a mechanism to control PHR1 activity. Whether this is the case for some other PHR1‐dependent genes is a question that should be evaluated in *phr1* knockout plants stably complemented with the wild type or the S11A variant of PHR1. These experiments are ongoing.

## AUTHOR CONTRIBUTIONS

RTF and PC conceived and designed the experiments. RTF, IRC, and AAC performed and analyzed the experiments. RTF and JAJD performed BiFC experiments. RRS designed, interpreted, and performed in silico experiments. EMB and PC were involved in data analysis and interpretation. PC wrote the manuscript. All authors read and approved the final manuscript.

## Supporting information


**Appendix S1.** Supporting information

## Data Availability

All data supporting the results shown in this study are included in the article and in its Supplementary data.
