# Supplementary material for: Phosphorylation of S11 in PHR1 negatively controls its transcriptional activity
Source: Physiol Plant. 2022 Dec 23;174(6):e13831. doi: 10.1111/ppl.13831 (PMC10107491; doi:10.1111/ppl.13831)
Supplement: Supplementary file 1 — Appendix S1. Supporting information [file PPL-174-0-s001.pdf]

Phosphorylation of S11 in PHR1 negatively controls its transcriptional activity

Ricardo Trejo-Fregoso[1], Iván Rodríguez[1], Alejandra Ávila[1], Javier Andrés Juárez-Díaz[2], Rogelio Rodríguez-Sotres[1], Eleazar Martínez-Barajas[1] and Patricia Coello[1]*

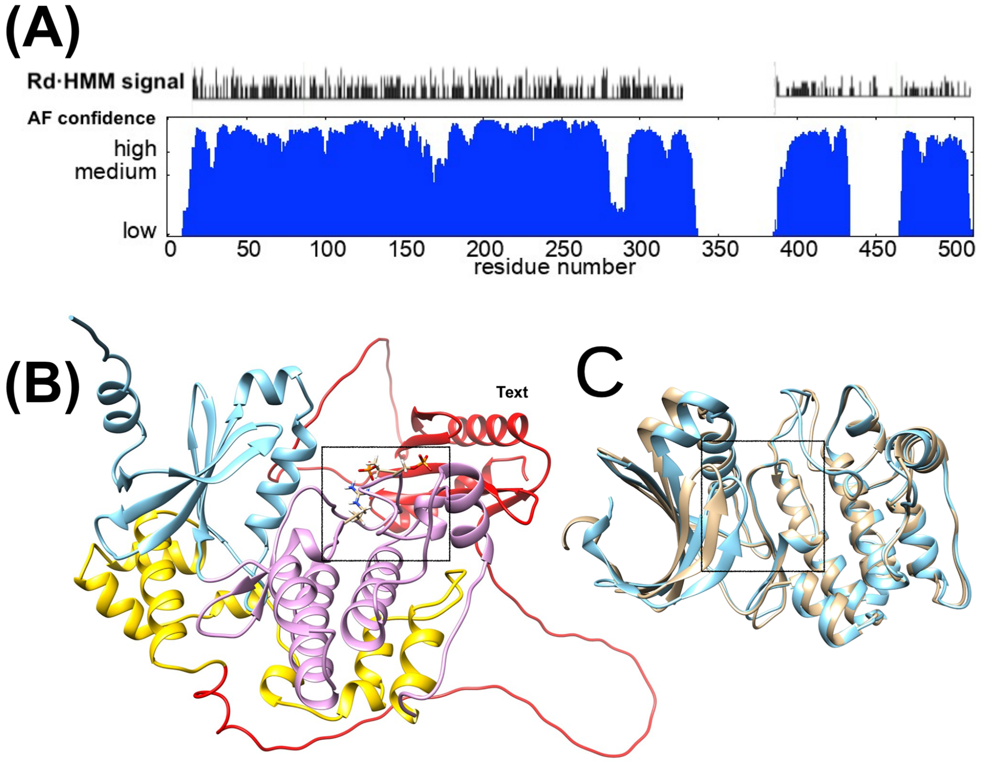

Figure S1. Evaluation of the three-dimensional structure of SnRK1α1 predicted by AlphaFold 2.0. **A**) The Rd·HMM probabilistic signal abbreviation for each residue (blank, + sign, lowercase, or uppercase amino acid) was converted to a 4-level bar and presented above the corresponding AlphaFold 2.0 confidence score. **B**) The folding pattern is shown as cartoon structures, colored by domain: N-terminal domain 1 in cyan; central all-α domain 2 in magenta; base domain 3 in yellow; and C-terminal domain 4 in red (including its intrinsically unstructured linker). **C**) Superposition of the catalytic cores of AURORA-A kinase (PDB ID 5OS1, shown as sky blue cartoon structures) with domains 1 and 2 of SnRK1α1 structural prediction (shown as light brown cartoon structures). The black squares enclose the zones included in Fig. 3. The molecular graphics were prepared with Chimera (Pettersen et al., 2004).

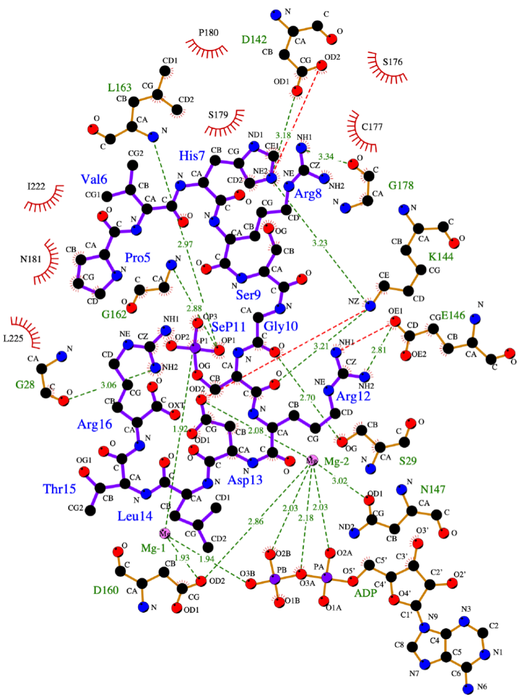

Figure S2. LigPlot of the complex between AtPHR1|5-16|Pser11 and SnRK1α1 ADP Mg^{2+} shown in Fig. 4A. Labels in blue with 3-letter amino acid codes correspond to the product peptide; labels with 1-letter amino acid codes correspond to enzyme residues making polar contacts (in green) or nonpolar interactions (in black). H-bonding distances are shown as green labels on green dashed lines, while detected salt bridges are shown as red dashed lines. The plot was prepared with LigPlot+ (Laskowski and Swindells, 2011). Lys 144 is indicated by an orange arrow, and P180 is indicated by a brown arrow.

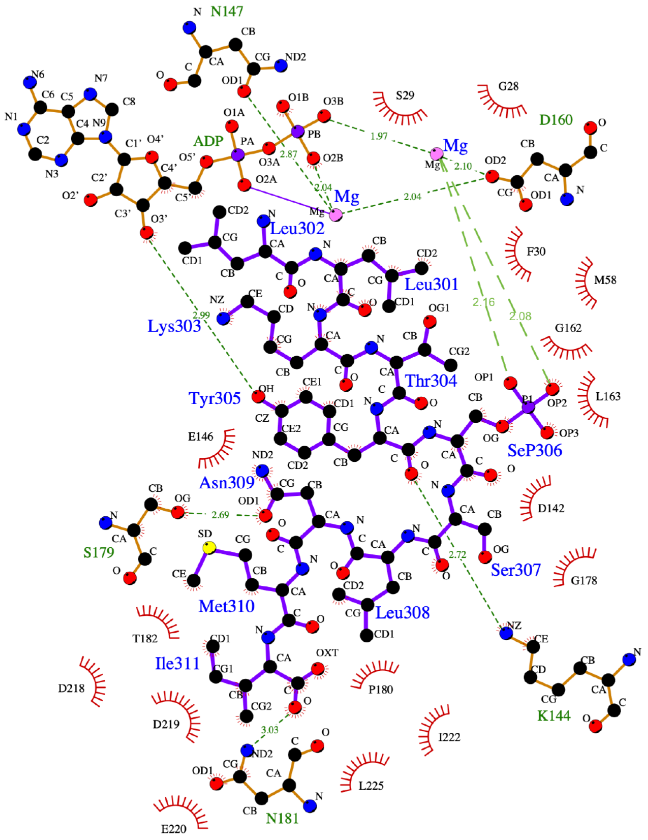

Figure S3. LigPlot of the complex between AtPHO1|301-316|Pser306 and SnRK1α1 ADP Mg^{2+} shown in Fig. 4B. Labels in blue with 3-letter amino acid codes correspond to the product peptide; labels with 1-letter amino acid codes correspond to enzyme residues making polar contacts (in green) or nonpolar interactions (in black). H-bonding distances are shown as green labels on green dashed lines, while detected salt bridges are shown as red dashed lines. The plot was prepared with LigPlot+ (Laskowski and Swindells, 2011). Lys 144 is indicated by an orange arrow, and P180 is indicated by a brown arrow.

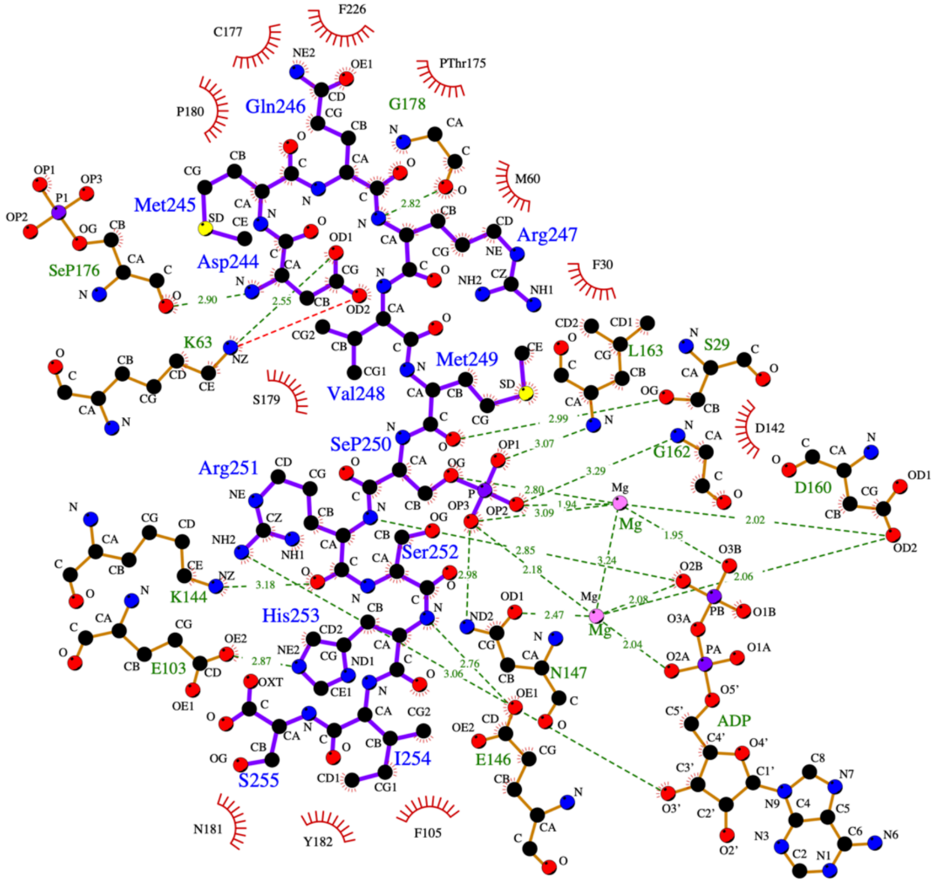

Figure S4. LigPlot of the complex between AtPHT1;8|244-255|Pser250 and SnRK1α1 ADP Mg^{2+} shown in Fig. 4C. Labels in blue with 3-letter amino acid codes correspond to the product peptide; labels with 1-letter amino acid codes correspond to enzyme residues making polar contacts (in green) or nonpolar interactions (in black). H-bonding distances are shown as green labels on green dashed lines, while detected salt bridges are shown as red dashed lines. The plot was prepared with LigPlot+ (Laskowski and Swindells, 2011). Lys 144 is indicated by an orange arrow, and P180 is indicated by a brown arrow.

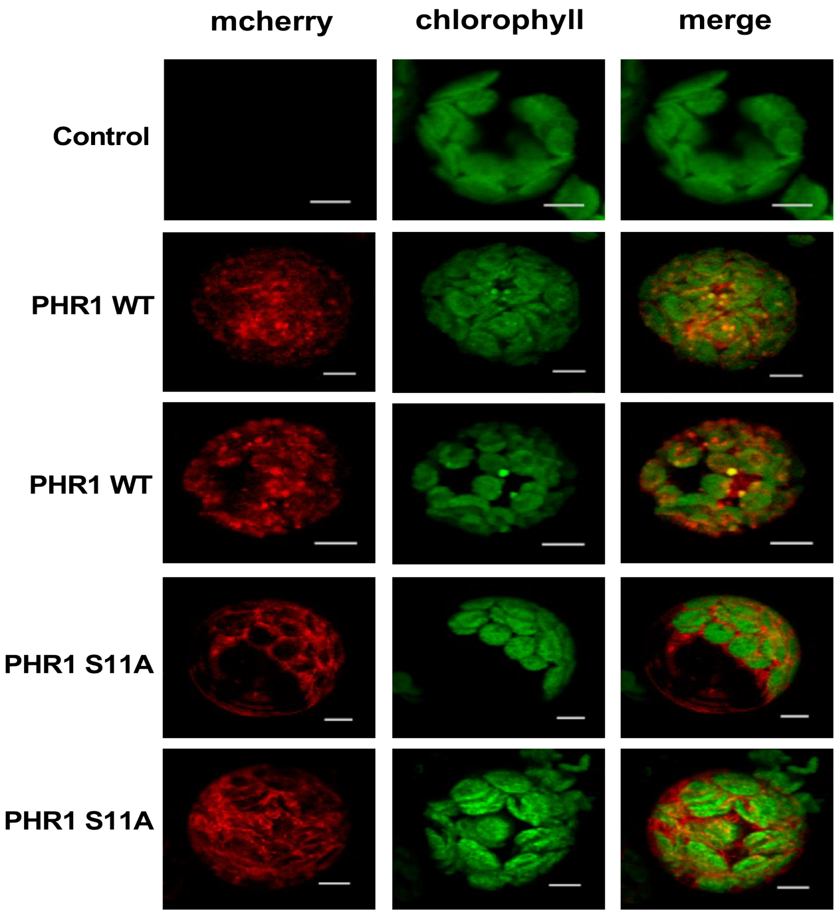

Figure S5. Expression of PHR1 WT and PHR1 S11A under +Pi and -Pi conditions. Transient expression of mCherry-PHR1 and mCherry-PHR1S11A in Arabidopsis protoplasts. After transfection, protoplasts were incubated in +Pi or -Pi medium (see Materials and Methods) for 16 h. Localization was detected using a confocal microscope FV3000 (Olympus). Scale bars: 15 μm.

Table S1. Peptide binding enthalpies form QM-PM7-LMO semiempirical calculations with implicit solvation.

Peptide	ΔH of formation† (kJ/mol)	ΔSAS-COSMO‡ (Å²)
AtPHR1|5-16|Pser11	-576.45	-941.9
AtPHO1|301-316|Pser306	-432.61	-919.2
AtPHT1;8|244-255|Pser250	-719.38	-1139.7

†The energy is calculated form the difference in heat of formation for the SnRK1α1-peptide complex minus the sum of heats of formation of SnRK1α1 and the free peptide.

‡The change in solvent accessible surface (SAS) is related to an increase in solvent (water) Entropy.
